# Umbilical Cord Blood Platelet Lysate as Serum Substitute in Expansion of Human Mesenchymal Stem Cells

**DOI:** 10.22074/cellj.2017.4886

**Published:** 2017-08-19

**Authors:** Negin Shirzad, Sima Bordbar, Alireza Goodarzi, Monire Mohammad, Pardis Khosravani, Froughazam Sayahpour, Mohamadreza Baghaban Eslaminejad, Marzieh Ebrahimi

**Affiliations:** 1 Department of Regenerative Biomedicine, Cell Science Research Center, Royan Institute for Stem Cell Biology and Technology, ACECR, Tehran, Iran; 2 Department of Stem Cells and Developmental Biology, Cell Science Research Center, Royan Institute for Stem Cell Biology and Technology, ACECR, Tehran, Iran

**Keywords:** Human Platelet Lysates, Umbilical Cord Blood, Peripheral Blood, Mesenchymal Stem Cell, Growth Factor

## Abstract

**Objective:**

The diverse clinical applications for human mesenchymal stem cells (hM- SCs) in cellular therapy and regenerative medicine warrant increased focus on developing adequate culture supplements devoid of animal-derived products. In the present study, we have investigated the feasibility of umbilical cord blood-platelet lysate (UCB-PL) as a standard substitute for fetal bovine serum (FBS) and human peripheral blood-PL (PB-PL).

**Materials and Methods:**

In this experimental study, platelet concentrates (PC) from UCB and human PB donors were frozen, melted, and sterilized to obtain PL. Quality control included platelet cell counts, sterility testing (viral and microbial), total protein concentrations, growth factor levels, and PL stability. The effects of UCB-PL and PB-PL on hMSCs proliferation and differentiation into osteocytes, chondrocytes, and adipocytes were studied and the results compared with FBS.

**Results:**

UCB-PL contained high levels of protein content, platelet-derived growth factor-AB (PDGF-AB), and transforming growth factor (TGF) compared to PB-PL. All growth factors were stable for at least nine months post-storage at -70˚C. hMSCs proliferation enhanced following treatment with UCB-PL. With all three supplements, hMSCs could differentiate into all three lineages.

**Conclusion:**

PB-PL and UCB-PL both were potent in hMSCs proliferation. However, PB promoted osteoblastic differentiation and UCB-PL induced chondrogenic differentiation. Because of availability, ease of use and feasible standardization of UCB-PL, we have suggested that UCB-PL be used as an alternative to FBS and PB-PL for the cultivation and expansion of hMSCs in cellular therapy.

## Introduction

The therapeutic potential of human mesenchymal stem cells (hMSCs) has been demonstrated by numerous clinical trials (1). Their differentiation potential into adipocytes, cartilages, and osteocytes support treatment of bone and cartilage diseases, their immune modulating role, increased therapeutic approaches for the treatment of autoimmune diseases, as well as the reduction of graft-versus-host responses (2- 4). The diverse clinical applications for hMSCs in cellular therapy and regenerative medicine merit an increased focus on developing culture methods devoid of animal-derived products (5-7). Usually, fetal bovine serum (FBS) is used to expand the majority of cells, including hMSCs, to promote cell growth and survival (8). However, because of its undefined growth factors and composition, the risk of contamination, batch-to-batch variation, xenogeny antigens and trigger of the immune response, animal welfare concerns and cost (7, 9, 10), its usage is limited in clinical-grade ex vivo expansion (11-13). To replace FBS as a growth supplement, recent research has focused on the application of serum-free (SF) media (14) or other human substitutes such as plateletderived products (5, 15-18). Intracellular granules of platelets consist of various reparative and mitogenic substances for hMSCs and fibroblasts that accelerate wound repair (19). Although autologous platelets provide safe supplements to treat diseases, the use of autologous platelets limits the amount of platelet lysate (PL) and therefore they are not sufficient for large-scale expansion. Batch-to-batch variability occurs following preparation of PL from small amounts of platelets. Therefore, finding an approach or substitute to minimize variability and produce a large amount of standard PL sufficient for several clinical scale expansions is necessary. In the present study, we have introduced PL from umbilical cord blood (UCB) as a cost-effective alternative for FBS or autologous PL and investigated its effect on hMSCs growth and differentiation

## Materials and Methods

### Production of platelet lysate from umbilical cord blood and peripheral blood

Healthy UCB samples and platelet concentrates (PC) were obtained from Royan Public Cord Blood Bank, Tehran, Iran and the Iranian Blood Transfusion Organization, respectively. We used the platelet rich plasma (PRP) method optimized in our laboratory to obtain PL from cord blood. Briefly, approximately 3-4 UCB samples were pooled in a 450 mL transfer bag (Besat Ind. Co., Iran) and centrifuged (300 g at 20˚C) for 22 minutes (acceleration: 5, deceleration: 0). This step, named as light spin, led to PRP separation. We gently collected PRP by a plasma extractor, which was centrifuged again at 5000 g (hard spin) at 20˚C for 30 minutes (acceleration: 5, brake: 2). Platelets precipitated firmly at the bottom of the blood bag and approximately 50 mL of plasma remained on the platelets, which we named PC. PCs from both UCB and peripheral blood (PB) were counted by an automated hematology analyzer (Sysmex Corporation, Kobe, Japan) and reached the appropriate platelet concentration (range: 1-5×10^9^ platelets/ml). For PL production from both UCB and PB, PCs were kept at -70˚C overnight and quarantined to test for the following infectious diseases: HIV-I/II, HCV, HBV, CMV, and HTLV-I/II, in addition to microbial sterility tests. Subsequently, frozen PCs were completely thawed in a 37˚C water bath and centrifuged at 3000 g for 30 minutes at 4˚C to remove platelet bodies. The resultant PL was aliquoted and stored at -80˚C until use.

### Measurement of protein concentration and growth factor content

We used the Bradford procedure to assay total proteins. Briefly, we generated a convenient standard curve using gamma globulin (range: 0-50 µg/µl) with Bradford reagent (Coomassie brilliant blue G); the OD was read at 595 nm with an ELISA reader (Multiskan Spectrum Microplate Spectrophotometer, Thermo Scientific, USA). The concentrations of platelet-derived growth factor- AB (PDGF-AB), transforming growth factor- beta 1 (TGF-β1), insulin growth factor-I (IGF-I), and basic fibroblast growth factor (bFGF) in PL were assessed by ELISA kits (all from R&D Systems, Minneapolis, MN, USA) according to the manufacturer’s instructions. 

### Stability of umbilical cord blood-platelet lysate

In order to assay for stability, different batches of UCB-PL were stored at -70˚C for nine months after which we analyzed the concentrations of PDGF- AB, TGF-β1, IGF-I, and bFGF. Because the freeze/ thaw cycles affect the absolute concentrations of cytokines, we processed the samples in such a way that they did not differ in the number of freeze/ thaw cycles. 

### Mesenchymal stem cell culture and proliferation assay

hMSCs were obtained from Royan Cell Bank, Tehran, Iran. A total of 5000 passage-3 hMSCs were seeded into six-well plates and cultured with minimum essential medium alpha (MEM-α 1X, Gibco, Invitrogen, Auckland, NZ) supplemented with 1% penicillin/streptomycin, and 1% 100X L-glutamine 200 mM (Gibco, Invitrogen, Auckland, NZ). Different concentrations of UCBPL (range: 1-30%) of 1-2×109 cells/ml of platelets and different numbers of platelets in each batch (range: 1-5×10^9^ platelets/ml (at the 10% dose) were added to the culture medium. SF medium was used as the negative control and 10% FBS was the positive control. hMSCs were counted by the MTS assay at 24, 48 and 72 hours post-treatment. In a separate experiment, 5 and 10% concentrations of both UCB-PL and PB-PL (1-2×10^9^ platelets/ml) were used to compare the two PL sources.

### Immunophenotyping of expanded human mesenchymal stem cells

We directly labeled 1×10^6^ cells with anti-human CD90-fluorescein isothiocyanate (FITC), CD105- phycoerythrin (PE), CD73-PE, CD44-PE, CD34- PE, and CD45-FITC in the dark for 20 minutes. Then, cells were washed in PBS (pH=7.4). As negative controls, cells were stained with FITC and PE-conjugated isotype controls. The specific fluorescence of 20000 cells was analyzed by FACSCalibur (Becton Dickinson, Temse, Belgium) using WinMDI 2.9 software.

### Adipogenic, osteogenic and chondrogenic differentiation

Passage-3 hMSCs were subjected to adipogenic, chondrogenic and osteogenic differentiation based on methods previously developed in our laboratory (20,21). For osteogenic differentiation, hMSCs in the presence of 10% FBS, 10% PB-PL, and 10% UCB-PL were induced by three weeks of culturing in DMEM that contained 50 mg/ml ascorbic acid 2-phosphate, 10 nM dexamethasone, and 10 mM β-glycerol phosphate. We confirmed differentiation by observation of extracellular matrix calcification with alizarin red staining. For adipogenic differentiation, we used DMEM-high glucose supplemented with 50 μg/mL indomethacin, 100 nM dexamethasone, and 50 μg/ml ascorbic acid 3-phosphate. Media were changed every three days. After three weeks, cells were fixed with cold 10% formalin for 1 hour, then washed twice with water and stained with an oil-red solution for 2 hours at room temperature in order to show the presence of intra-cellular lipid droplets in the cytoplasm. Cells were washed twice and observed under an optical microscope. For chondrogenic differentiation, 2.5×10^5^ from passage-3 hMSCs in three groups were pelleted by centrifugation at 300 g for 5 minutes. Then, cells were cultured in DMEM supplemented with 10 mg/mL TGF-β3, 10 mg/mL bone morphogenetic protein-6 (BMP6), 50 mg/mL insulin transferrin selenium+premix, and 1.25 mg bovine serum albumin (all from Sigma- Aldrich, Deisenhofen, Germany) for three weeks. 

### Real-time quantitative polymerase chain reaction

Cells were harvested, after which we isolated total RNA with an RNA extraction kit (TaKaRa, Japan). We used 100-500 ng of total RNA for reverse transcription with the Prime Script II Strand cDNA Synthesis Kit (TaKaRa, Japan). Polymerase chain reaction reactions were run in duplicate using 1/40^th^ of the cDNA per reaction and 400 nM forward and reverse primers with a SYBR Green master mix (TaKaRa, Japan) in the Rotor Gene 3000 (Corbett Research). Quantitative RTPCRs were performed in duplicate for each sample primer set and we considered the mean of the three experiments as the relative quantification value. Relative gene expression was analyzed using the comparative Ct method, 2^-ΔΔCt^. All samples were normalized to the levels of *GAPDH*, as the loading control. Table 1 lists the primer sequences.

### Statistical analysis

We performed all of the experiments at least in triplicate. Data have been presented as mean ± SD. The data were analyzed by one-way ANOVA and non-parametrically validated by the Wilcoxon 406 signed rank test. Values of P≤0.05 were considered significant. 

## Results

**Fig.1:**
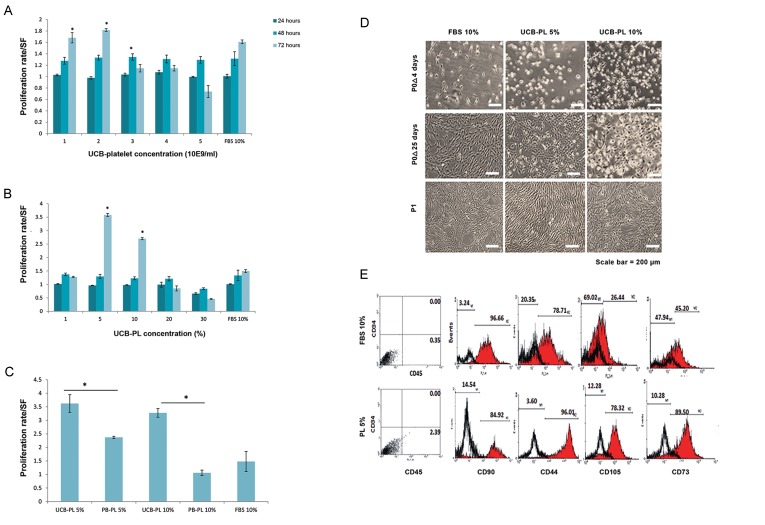
Human mesenchymal stem cell (hMSC) characterization. A. We used different number of platelets to produce umbilical cord blood platelet lysate (UCB-PL) which were added to the hMSC culture medium at a 10% dose in order to determine the best cell concentration that produced standard PL, B. 1-2×10^9^ platelets/ml were used to prepare UCB-PL and added to hMSC medium at concentrations that ranged from 5 to 30% to find the effective dose of UCB-PL on hMSC proliferation, C. 1-2×10^9^ platelets/ml of cord blood and PB were used to prepare PL and added to hMSC medium at doses of 5 and 10% to compare their effect on hMSC growth, D. Morphology of hMSCs cultured with UCB-PL or 10% fetal bovine serum (FBS) as the positive control, as assessed by light microscope, and E. Immunophenotype of hMSCs cultured in the presence of UCB-PL or FBS. The experiments were performed for three replicates. *; P≤0.05, PB-PL; Peripheral blood-platelet lysate, and SF; Serum-free.

hMSCs showed increased proliferation in UCBPL compared to PB-PL and FBS. We performed two types of experiments to understand the effect of UCB-PL on hMSC proliferation. First, we used 1-5×10^9^ cells/ml platelets for UCB-PL production and added the 10% dose to the culture medium. In the second experiment, UCB-PL with a cellular concentration of approximately 1-2×10^9^ cells/ml was prepared and added to the culture medium at different concentrations (1, 5, 10, 20, 30%). As shown in Figure 1A and B, the best platelet count for production of UCB-PL was 1-2×10^9^ cells/ml that showed significant enhancement of hMSC proliferation compared to 10% FBS as the control group (P≤0.01). The 5 and 10% doses of UCB-PL showed maximum effects on MSCs proliferation (P≤0.05, Fig .1B). In both experiments, the higher number of platelets previous UCB-PL production or higher concentrations of UCB-PL caused to dramatically reduction in MSCs which may be due to cell cytotoxicity (Fig .1A, B). A comparison of both types of PL (derived from cord blood or PB) showed that UCB-PL significantly increased the numbers of hMSCs compared to PB-PL (P≤0.02, Fig .1C). Although the morphology of hMSCs cultured in the presence of UCB-PL and FBS was the same (Fig .1D), they had significantly different expression patterns of hMSC related markers, particularly CD44, CD105, and CD73 (Fig .1E).

**Table 1:** Primer sequences and characteristics


Specificity	Gene name	Primer sequence (5´-3´)	Annealingtemperature ( °C)

Bone	*OSTEOCALCIN*	F: GGCAGCGAGGTAGTGAAGAG	61
		R: CAGCAGAGCGACACCCTAGAC	
	*RUNX2*	F: ATGACACTGCCACCTCTGA	60
		R: ATGAAATGCTTGGGAACTGC	
	*ALP*	F: CAACAGGGTAGATTTCTCTTGG	60
		R: GGTCAGATCCAGAATGTTCC	
Adipose	*PPAR GAMA*	F: TCTCCAGCATTTCTACTCCACA	60
		R: GATGCAGGCTCCACTTTGAT	
	*ADIPONECTIN *	F: CCTGGTGAGAAGGGTGAGAA	60
		R: CAATCCCACACTGAATGCTG	
	*LPL*	F: TCAACTGGATGGAGGAGGAG	60
		R: GGGGCTTCTGCATACTCAAA	
Chondrocyte	*COL2*	F: TCTACCCCAATCCAGCAAAC	58
		R: GCGTAGGAAGGTCATCTGGA	
	*SOX9*	F: CCCTTCAACCTCCCACACTAC	60
		R: GCTGTGTGTAGACGGGTTGTT	
	*AGGRECAN *	F: CTGGACAAGTGCTATGCCG	58
		R: GAAGGACCGCTGAAATGC	
	*GAPDH*	F: CTCATTTCCTGGTATGACAACGA	60
		R: CTTCCTCTTGTGCTCTTGCT	


### The effect of umbilical cord blood-platelet lysate and peripheral blood-platelet lysate on human mesenchymal stem cell differentiation 

**Fig.2:**
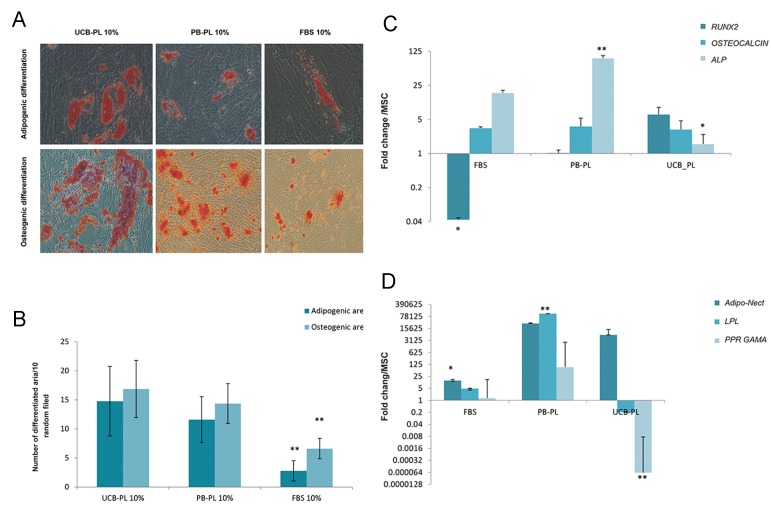
Comparison the effects of umblical cord blood-platelet lysate (UCB-PL), peripheral blood-platelet lysate (PB-PL) and fetal bovine serum (FBS) on mesenchymal stem cell (MSCs) differentiation. A. Pretreated MSCs in osteogenic and adipogenic differentiation medium supplemented with 10% UCB-PL, PB-PL and FBS which were subsequently counterstained with alirizan blue and oil red to specify differentiation, B. To quantify the results, ten filed randomly were selected and differentiation area counted in both PL groups, C. Expression of adipocyte genes, D. Osteocyte genes as assessed by real-time quantitative polymerase chain reaction (RT-PCR), which showed that PB-PL increased expression of the differentiation genes. *; P<0.05 and **; P<0.01.

Passage-3 hMSCs were pre-expanded in 10% UCB-PL and 10% PB-PL as the test groups, and 10% FBS as the control group. These hMSCs were induced to differentiate into osteogenic, adipogenic and Chondrogenic cells as described in Materials and Methods. In order to quantitate data, we randomly counted 10 fields from each group. After three weeks of treatment with defined medium for osteogenic and adipogenic differentiation, we observed calcium depositions and lipid droplets in all groups when counterstained with Oil red and Alizarin blue, respectively (Fig .2A). Quantification of data showed significantly higher mineral deposition and lipid droplets in the UCB-PL and PB-PL groups compared to FBS (P≤0.001, Fig .2B). Although there were no differences between UCB-PL and PB-PL in numbers of differentiated regions (P≥0.7) in both adipogenic and osteogenic differentiation, the differentiation region was morphologically larger in UCB-PL (Fig .2A). 

### Expression patterns of osteogenic, adipogenic and Chondrogenic marker genes

We analyzed expressions of specific markers both before and during trilineage differentiation. For osteogenic differentiation, we chose runt-related transcription factor 2 (*RUNX2*) as an early marker of differentiation, alkaline phosphatase (*ALP*) as a continually expressed marker, and *OSTEOCALCIN* as a late marker of differentiation. As shown in Figure 2C, through the course of osteogenic differentiation, *RUNX-2* expression down- regulated in the FBS group (P=0.008) and up- regulated in the UCB-PL and PB-PL groups. *RUNX-2* up-regulation was dominant in the PB- PL (P=0.01) group. *OSTEOCALCIN* expression significantly increased (P≤0.05) in all groups with no differences observed between the groups (P≥0.05), which was an expected finding (Fig .2C). Differences in *ALP* expression could be attributed to culture supplements, which significantly up-regulated in PB-PL compared to the other groups (P≤0.004). For adipogenic differentiation, we chose *PPAR gamma* and *ADIPONECTIN* as specific markers for early differentiation and *LPL* for later expression. As shown in Figure 2D, hMSCs cultured in the presence of PB-PL showed significant up-regulation in the selected adipogenic- specific markers (P≤0.02). For chondrogenic differentiation, we selected *SOX9, AGGRECAN,* and *COL2* according to the differentiation step. *SOX9* expressed during early differentiation, whereas *AGGRECAN* and *COL2* expressed late in the differentiated cells. SOX9 down regulated in the UCB-PL group (P≤0.02) and AGGRECAN up-regulated significantly. COL2 increased in all groups, but was dominant in the PB-PL group (Fig .2D). 

### Comparison of growth factor content in umbilical cord blood-platelet lysate and peripheral blood- platelet lysate 

The concentration of important growth factors in UCB-PL was tested by ELISA in eight different batches and compared with PBPL at the same platelet concentration (1-2×10^9^/ ml). As shown in Table 2, the concentration of TGF-β1, IGF-1, and PDGF-AB was had higher we observed significantly higher concentrations of compared to the PB-PL group (P≤0.004). The concentration of bFGF was not significant between groups (P=0.8). There was significantly higher in the UCB-PL group compared to the PB-PL group at the same platelet concentration. We assessed stability of PDGF-AB as the main growth factor for hMSCs, TGF-β, IGF, and bFGF nine months after freezing at -20˚C. The majority of proteins from all samples ranged from approximately 90 to 100 mg/ml. The results determined that the concentration of all tested growth factors were the same as the prefrozen values (P≥0.05, Fig .2). However, their potential should be checked in order to confirm stability.

**Table 2:** Concentration of major growth factors in umbilical cord blood-platelet lysate (UCB-PL) and peripheral blood platelet lysate (PB-PL)


Sample	bFGF (ng/ml)		TGF-β1(ng/ml)		IGF-1(ng/ml)		PDGF-AB (ng/ml)		Total protein (mg/ml)	
	UCB-PL	PB-PL	UCB-PL	PB-PL	UCB-PL	PB-PL	UCB-PL	PB-PL	UCB-PL	PB-PL

1	0.066	0.072	55.38	29.75	470.96	320.61	675.03	372.8	74.85	42
2	0.054	0.0737	51.36	31.39	436.00	322.36	627.13	463.4	65.33	30
3	0.057	0.040	56.64	38.59	505.93	343.34	638.29	332.6	73.65	55
4	0.063	0.059	58.204	27.98	575.86	327.60	553.81	318.8	62.45	44
5	0.054	0.045	51.63	18.85	558.38	313.62	580.02	392.6	91.225	45
6	0.066	0.044	ND	ND	487	ND	490.91	493.4	85.6	82
7	0.054	0.072	ND	ND	477.2	ND	480.63	367.8	67.8	46
Mean ± SD	0.059± 0.005	0.058± 0.014	56.04± 3.45	29.31± 7.102	501.62± 49.67	325.50± 11.153	577.97± 74.29	391.62± 64.83	90.46± 6.21	49.14 ± 16.25
P value	0.854		0.0008		0.002		0.004		0.0006	


bFGF; Basic fibroblast growth factor, TGF-β1; Transforming growth factor-beta1; IGF-1; Insulin growth factor-I, and PDGF-AB; Plateletderived growth factor-AB.

## Discussion

*Ex vivo* expansion of hMSCs, as a strong cell therapy candidate, requires the addition of supplements to basal culture medium. Most early clinical trials have used FBS in their expansion protocols (3, 22). However, because of safety concerns, non-animal alternatives are warranted (14). Human PL (hPL) is considered an alternative source in hMSCs cultures because of the role of platelets in attracting stromal cells to the injury site and promotion of wound healing (23, 24). Therefore, many studies have used autologous human plasma or PC in addition to expired platelets to determine their role in hMSC proliferation, migration, and differentiation (5, 25-27). Our approach was to provide a novel source of PL from cord blood that was accessible for all cord blood banks and had the capability to be standard for clinical scale expansions. Therefore, in this study we compared UCB-PL as a growth supplement for hMSCs proliferation and differentiation to PB-PL and the commonly used FBS. We used cord blood from donor mothers who had to fulfill stringent donor eligibility criteria, including negative results for infectious disease markers (HIV, HBC, HCV, HAV, and syphilis). In addition, cord bloods were tested for infectious diseases by PCR as well as for microbial contamination pre- and post-PL production. The production process of PL included a freeze-thaw process which would be helpful for clinical grade production. Our results determined that surface antigen expression in hMSCs remained unaltered. However, the use of UCB-PL as an hMSC growth supplement significantly increased proliferation in a dose-dependent manner. The effects of UCB-PL on hMSCs differentiation showed that some of the adipogenic, osteogenic, and chondrogenic specific genes down-regulated compared to PB-PL, however adipogenic and osteogenic differentiation of hMSCs pre-treated with UCB-PL and PB-PL remained the same. As mentioned above, hMSCs continually had faster proliferation in UCB-PL compared to PB-PL and FBS. This trend could be attributed to the higher growth factor content in UCB-PL compared to PBPL. Murphy et al. (26) reported a higher growth factor content in PRP derived from CB compared to adult PRP. The combination of PDGF, bFGF, TGF-β, and IGF-1, as mitogens for hMSCs, were sufficient for hMSCs expansion in a SF culture model under laboratory-scale conditions (28, 29). During trilineage differentiation, hMSCs underwent typical morphological changes from fibroblast-like cells to cells that had cuboidal shape and the capability of mineralization or formation of lipid droplets in all groups. Although hMSCs treated with UCB-PL and PB-PL were more potent in adipogenic and osteogenic differentiation, they differed in expressions of specific genes in each lineage of differentiation.

The cells propagated in PB-PL tended to express osteogenic genes more than cells propagated in FBS or UCB-PL. The main difference was in ALP activity as a marker of osteoblastic cells actively involved in the mineralization process. In this group, we have observed *RUNX2* expression at a level much lower than in UCB-PL but at a higher level than FBS. *RUNX2* is a transcription factor that has high levels of expression during early osteogenesis and an indicator of good quality osteogenic differentiation (5). Although both UCB-PL and PB-PL could differentiate hMSCs into osteoblasts when stained with alizarin red, PBPL was an appropriate supplement when the cells were directed toward bone cell lineages. Similar effects have been observed for human and murine osteoblasts and ligament cells cultured with PRP. In terms of chondrogenic differentiation, the cells propagated in UCB-PL appeared to have expressed a higher level of the cartilage specific gene, aggrican (the proteoglycan aggregate responsible for the characteristic biomechanical property of hyaline cartilage) (30). This data indicated that, for cartilage application, hMSCs could preferentially be propagated in a medium supplemented with UCB-PL rather than FBS. Our data revealed that with the same number of platelets used to produce PL, UCB-PL contained higher levels of PDGFAB, IGF1, and TGF-β1 compared to PB-PL as previously reported by Murphy et al. (26). We have shown that TGF-β, bFGF, and PDGF-AB which are commonly used in cell culture media maintained stability at -70˚C for at least nine months. Therefore, we have designed a very safe human medium supplement. One may consider the use of autologous PL. However, most pB-PL donors are ill or have a chronic disease. Harvesting BM or PC may be difficult. When the allogenic sources have been used, batch to batch variation increases because of the requirements for high amounts of culture medium for the hMSC culture. Therefore, the substantial benefits of UCB-PL in the proliferation and differentiation of hMSCs have been validated in this study.

## Conclusion

Our study showed that the UCB-PL could be easily produced in a cord blood bank as a costeffective product with lower batch to batch variation. As discussed above, the closely fetal nature of UCB provided a rich and unique combination of nourishing factors in addition to high levels of molecules such as PDGF, TGF and IGF1. This collectively suggested that UCBPL might present a viable alternative to FBS and autologous PL for the propagation of hMSCs in culture. Future studies should be planned to determine the presence of chromosomal instability or immunomodulation of hMSCs cultured for an extended period in UCB-PL.
